# Crystallization of a Self-Assembling Nucleator in Poly(l-lactide) Melt

**DOI:** 10.1021/acs.cgd.1c00750

**Published:** 2021-09-01

**Authors:** Wei Wang, Angelo Saperdi, Andrea Dodero, Maila Castellano, Alejandro J. Müller, Xia Dong, Dujin Wang, Dario Cavallo

**Affiliations:** † Department of Chemistry and Industrial Chemistry, University of Genoa, Via Dodecaneso 31, Genova 16146, Italy; ‡ POLYMAT and Department of Polymers and Advanced Materials: Physics, Chemistry and Technology, Faculty of Chemistry, University of the Basque Country UPV/EHU, Paseo Manuel de Lardizabal, 3, 20018 Donostia—San Sebastian, Spain; § IKERBASQUE, Basque Foundation for Science, Bilbao 48009, Spain; ∥ Beijing National Laboratory for Molecular Sciences, Institute of Chemistry, Chinese Academy of Sciences, Beijing 100190, China; ⊥ University of Chinese Academy of Sciences, Beijing 100049, China

## Abstract

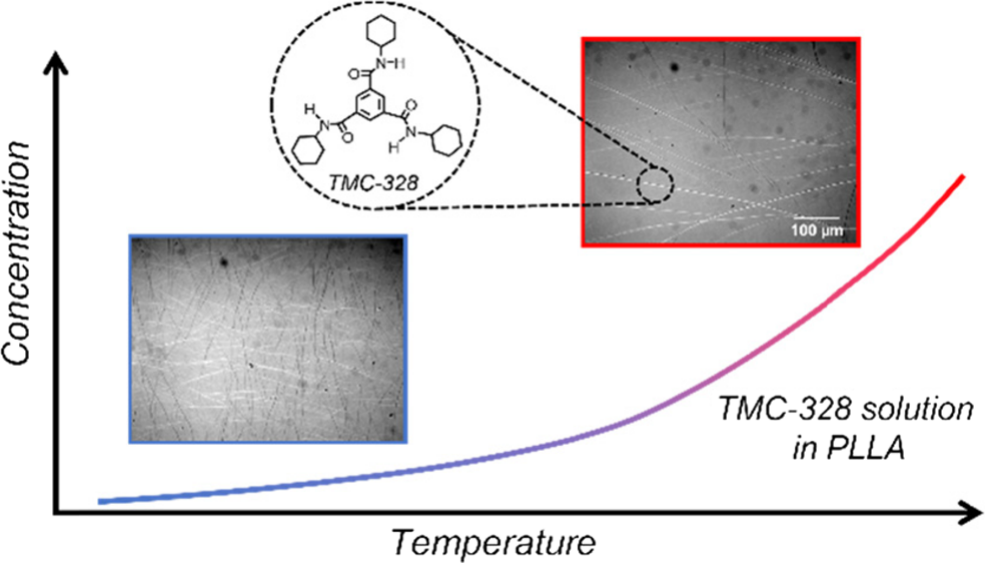

In the present work, crystallization of a soluble nucleator *N*, *N*′, *N*″-tricyclohexyl-1,3,5-benzenetricarboxylamide (TMC-328) in a poly(l-lactic acid) (PLLA) matrix has been studied at different temperatures. Based on the change in solubility with temperature, different levels of supersaturation of TMC-328 in a PLLA matrix can be obtained. This nucleator presents a fibrous structure produced via self-assembling and develops into an interconnected network when the temperature is lowered. The TMC-328 crystal nuclei density is quantified via optical microscopy, using the average distance of the adjacent fibrillar structure, which shows a steady decrease with the decrease in temperature. The crystallization rates of TMC-328 were assessed through rheological measurements of network formation. Both fibrils’ density and crystallization kinetics display a power law dependence on supersaturation. For the first time, the solid–melt interfacial energy, the size of the critical nucleus, and the number of molecules making up the critical nucleus of the nucleator TMC-328 in the PLLA matrix have been determined by adopting the classical nucleation theory. The subsequent crystallization of PLLA induced by this nucleator was investigated as a function of the fibrils’ spatial density. The crystallization rate of PLLA is enhanced with the increase in the TMC-328 fibrils’ density because of the availability of a larger nucleating surface. The self-assembled fibril of TMC-328 can serve as shish to form a hybrid shish-kebab structure after the crystallization of PLLA, regardless of the number of nucleation sites.

## Abstract 2

The equilibrium solubility of a nucleating agent (TMC-328) in poly(l-lactide) melt is determined as a function of temperature. Crystallization experiments at different supersaturations, above the melting point of the polymer, are then performed. It is shown that both the spatial density of fibrillar crystals and the overall crystallization rate of TMC-328 are strong functions of the degree of supersaturation. The results are in agreement with the classical nucleation theory of small molecular crystals.

## Introduction

Nucleating agents (NAs) are typically used when crystallization is particularly slow as in the case of poly(ethylene terephthalate) (PET) or to optimize property combinations for specific applications such as for isotactic polypropylene (iPP).^1−5^ Apart from the two thermoplastics already mentioned, nucleation additives are mainly relevant for various polyamides (PA-6 and PA-66) and other polyesters such as poly(butylene terephthalate) (PBT) and poly(l-lactic acid) (PLLA). It has been proposed that many materials, such as talc, montmorillonite, carbon nanotubes, and some inorganic powders, have a strong ability to induce nucleation of the abovementioned materials.^6−18^ The main problem is the difficulty in dispersing these typically inorganic additives in polymer matrices. Indeed, to act optimally, these NAs need to be dispersed as homogeneously as possible. Obtaining sufficient dispersion is technologically challenging, and to achieve this, a new class of additives that are soluble in molten polymers has been introduced.^19−25^

These soluble NAs are organic, and at high temperatures, they dissolve completely in the polymer melt. During cooling, because of the decrease in solubility, the active molecules undergo phase separation to form networks of fibrils with a high nucleating capacity toward the polymer. The formation of the network is due to the self-organization of the additive molecules that have a lyophilic part capable of solubilizing in the organic liquid (polymeric melt), while the other part is responsible for the establishment of relatively strong bonds (via hydrogen or ionic bonds, usually in a linear fashion), which is necessary for the growth of the microfibrillar structure.^26,27^

As the most concerned eco-friendly thermoplastic polyester, PLLA is increasingly being used in recent years because of its excellent biocompatibility and processability. However, its slow crystallization kinetics caused by the stiffness of the chains and the steric size of the methyl group cannot be neglected any longer.^28−31^ This has a strong impact on the processability and final characteristics of the product, limiting the possible practical applications.^31−36^ Based on past research, the addition of self-assembly NAs is the most probable effective way to improve the crystallization ability of PLLA. The nucleators, such as dibenzylidene sorbitol, aliphatic amides, benzenetricarboxylamide derivatives (TMC-328), tetramethylenedicarboxylic dibenzoylhydrazide (TMC-306), and octamethylenedicarboxylic dibenzoylhydrazide (TMC-300), have been utilized not only to enhance the overall crystallization rate but also to tune the crystalline morphology of PLLA.^35−42^ For example, Bai et al. found that *N*, *N*′, *N*″-tricyclohexyl-1,3,5-benzenetricarboxylamide (TMC-328) induces fast crystallization and shows noticeable effects on controlling the crystal morphologies of PLLA, including conelike, shish-kebab-like, and needle-like macroscopic structures, because of the high ability in self-organization.^36^ Indeed, all studies on PLLA/NA compounds only focus on the crystallization of PLLA after the self-assembling of nucleators and the interaction between them. Nevertheless, until now, the crystallization process of the NA within the PLLA matrix is unrevealed.

In the present work, we wanted to approach the self-organization of TMC-328 molecules in PLLA by comparing this process with the crystallization of organic molecules in solution. This process depends primarily on solubility, which is a function of temperature and concentration. In general, the solubility of a solute is represented by the solubility curve, a diagram showing the concentration as a function of temperature. The solubility curve divides the concentration and temperature range into two distinct zones, one of stability (unsaturated solution) and one of instability (supersaturated solution). Nucleation and growth phenomena depend on supersaturation, that is, the actual solution concentration with respect to the equilibrium one. Thus, the average final size and number of crystals present at the end of the crystallization process will depend on the coordinate of a given system on the solubility plot.^43−45^ At a low degree of supersaturation, crystal growth is faster than the nucleation rate, resulting in a larger particle size distribution. However, at a higher degree of supersaturation, crystal nucleation dominates crystallization, eventually resulting in the appearance of smaller crystals.^46,47^ This analogy has allowed us to explain the size of the NA network as the concentration and growth temperature are changed. Understanding and controlling the kinetics of the growth of the additive fibrils is crucial for predicting the final morphology of PLLA crystals.

## Experimental Section

### Materials

Samples of PLLA in the pellet form were kindly provided by Purac Biochem (Gorinchem, The Netherlands). The PLLA sample has a weight-average molar mass of 226 kg/mol and a melt flow index of 6.9 g/10 min, and it shows a nominal melting temperature of 175.4 °C. The NA TMC-328 was supplied by the Shanxi Provincial Institute of Chemical Industry (Co., Ltd.), China. The chemical structure is reported in the Supporting Information (Figure S1).

### Sample Preparation

Both NAs and PLLA pellets were dried at 50 °C for 24 h before melt-blending in a Brabender mixer. To achieve good dispersion of the NA in PLLA, mixing was performed at 190 °C and 60 rpm for 6 min. Moreover, for the sake of comparison, pure PLLA was also processed using the same method. We investigated the following concentrations of TMC-328 in PLLA: 0.1, 0.2, 0.3, 0.5, and 1 wt %. For simplicity, the samples are abbreviated as PLLA-*x*, where *x* denotes the weight percentage of TMC-328 in the polymer matrix.

### Thermogravimetric Analysis

Thermogravimetric analysis (TGA) was performed with a TGA 1 instrument (Mettler Tolendo). About 10 mg of PLLA and TMC-328 samples were used. The material was heated from 20 to 800 °C with a heating rate of 10 °C/min under nitrogen flow. The temperature of the maximum degradation rate for both PLLA and TMC-328 is about 300 °C, as shown in Figure S2 in the Supporting Information.

### Polarized Light Optical Microscopy

The crystallization process and crystal morphology of PLLA and TMC-328 were observed in situ using a Leica DMLP optical microscope under crossed polarizers, equipped with a computer-controlled digital camera (Optika B5). The PLLA–TMC 328 films were prepared by compressing the polymer blends on a hot stage at 200 °C. The thermal protocol adopted during experiments was controlled using a Linkam THMS600 hotstage. First, after dissolution at 240 °C for 3 min, a protocol with cooling and further heating at a rate of 5 °C/min was used to determine the point of precipitation and dissolution of the self-assembling structure. Second, in order to study the isothermal crystallization of TMC-328, followed by the nucleation of PLLA on its surface, the samples were cooled to the growth temperature of the network at 10 °C/min from the isotropic melt. Subsequently, the isotherm was maintained for 20 min, and then, the samples were cooled to room temperature at 5 °C/min.

### Rheological Analysis

The rheological behavior of all samples was characterized using a rotational shear rheometer (Discovery HR 10, TA Instruments) equipped with a parallel plate (PP) geometry with a diameter of 25 mm and approximately 1 mm gap. The rheometer was equipped with an environmental test chamber (ETC) that uses controlled convection/radiant heating and a constant flow of nitrogen to provide a fast and stable temperature response and prevent polymer degradation. The disk-shaped samples were prepared by compression molding at 200 °C and held at that temperature for 3 min to completely melt PLLA and TMC-328. Time sweep measurements at 175, 180, 185, 190, and 195 °C were performed to monitor the evolution of the storage modulus during the isothermal network formation of TMC-328 after dissolution at 240 °C for 3 min. The applied strain and oscillation frequency were 1% within the linear viscoelastic region (as verified via an amplitude sweep test) and 2 rad/s, respectively.

### Differential Scanning Calorimetry

The crystallization behavior of the samples was probed on a TA Instruments DSC 250 under the nitrogen atmosphere. For each measurement, around 5 mg of the sample was placed in a sealed aluminum pan. We applied the same protocol as adopted in the polarized light optical microscopy (POM) test to study the effect of the spatial density of the network on PLLA crystallization.

## Results and Discussion

### Crystallization Behavior of TMC-328 in the PLLA Matrix

**Figure 1:**
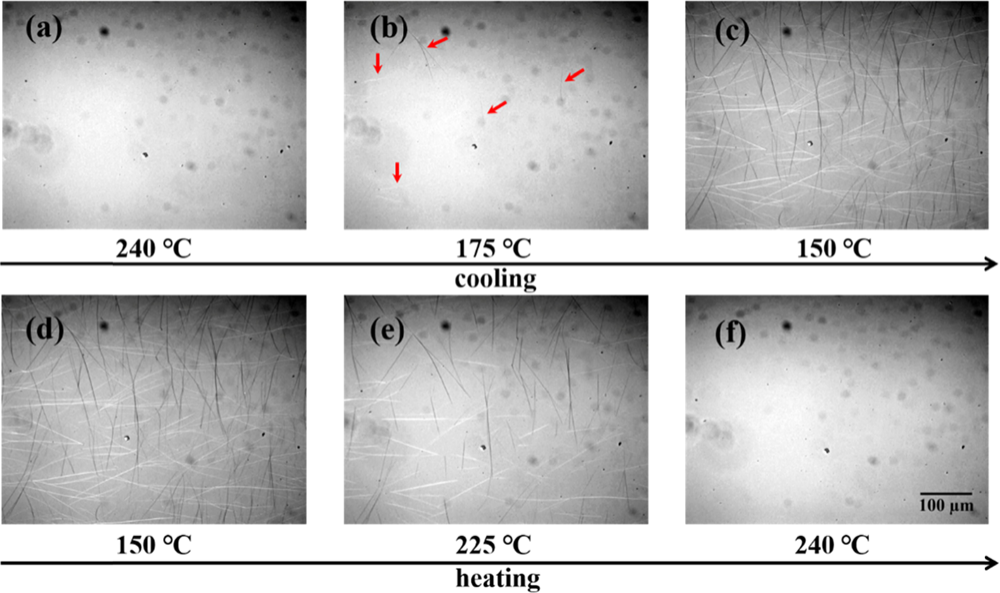
Polarized optical micrographs taken during self-assembling and melting of 0.3% TMC-328 in the PLLA matrix. The contrast of the images has been adjusted to enhance the visibility of the fibrils.

We first investigated the crystallization and melting behavior of nucleator TMC-328 dispersed in the PLLA matrix. An overview of the POM observation of the PLLA-0.3 sample upon cooling from the homogeneous melt and subsequent heating is shown in Figure 1. When the sample is annealed at 240 °C for 3 min, TMC-328 is dissolved in the PLLA melt, and a homogeneous mixture is obtained above the melting temperature of PLLA because no obvious birefringence of TMC-328 crystals is observed within the resolution scale of POM (Figure 1a). Upon cooling, the nucleator starts to crystallize into a fibrous structure with a length of several tens of micrometers at 175 °C, as indicated by the red arrows shown in Figure 1b. TMC-328 fibrils grow simultaneously from multiple sites in the latter stage of crystallization. In addition to the increase in the fibril length, fibrils also form branches through self-assembling (Figure 1c). Therefore, the resulting network formed by these fibrils is dense because of the connection between the fibrils and the increasing branching number. In the subsequent heating process (Figure 1d–f), the fibrils of TMC-328 formed during cooling crystallization dissolve gradually in the PLLA matrix, which is similar to a solute that dissolves in a solvent.

**Figure 2:**
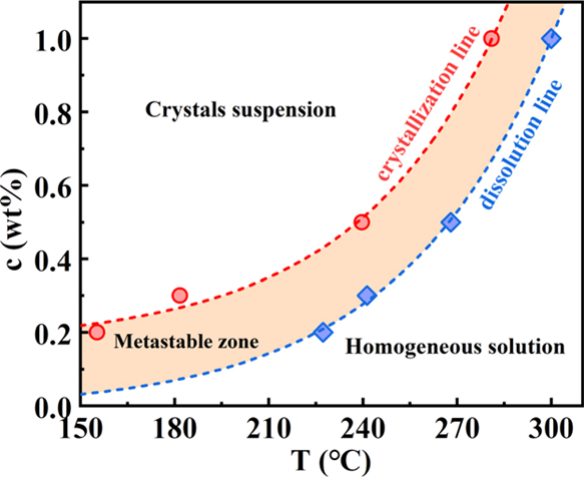
Dissolution and crystallization temperatures of TMC-328 in the PLLA matrix at various concentrations, as determined from POM. The dashed lines are exponential fitting curves.

In order to figure out the influence of concentration on the dissolution and self-assembling (crystallization) of TMC-328 in the PLLA matrix, we employed visual observation to judge the appearance and disappearance of crystals during cooling and heating at a rate of 5 °C/min by POM. The corresponding concentration (*c*)–temperature (*T*) diagram for dissolution and crystallization of TMC-328 is shown in Figure 2. It is clear that the crystallization and the dissolution behaviors of TMC-328 in the PLLA matrix strongly depend on its content. With the increase in the nucleator concentration (0.2–1 wt %), the crystallization temperature of TMC-328 increases from 155 to 281 °C during cooling, and the dissolution temperature increases from 227 to 300 °C, as displayed by the red and blue dashed lines in Figure 2. It should be noted that the blue line in Figure 2 can be regarded as the temperature dependence of the solubility (with equilibrium concentration, *c*_e_) of TMC-328 in the PLLA matrix. The stable solution can be observed in the region below the dissolution line (*c* < *c*_e_), indicating the complete dissolution of TMC-328 in PLLA. Cooling crystallization can occur when the solubility is sufficiently low (*c* > *c*_e_) at a certain temperature, that is, if supersaturation is realized. Between the dissolution line and the crystallization line, the metastable zone (MSZ, orange part) is recognized, in which homogeneous or heterogeneous nucleation occurs and crystals can grow by consuming the supersaturation. When the content of the nucleator is large enough (i.e., in the region above the crystallization line), nucleation is no longer difficult, and the solute rapidly precipitates.

**Figure 3:**
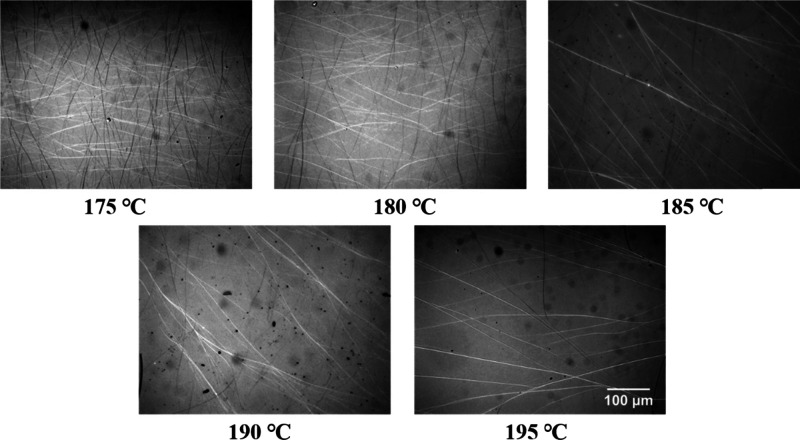
Polarized optical micrographs taken during isothermal crystallization of PLLA-0.3 at the indicated temperatures.

It is well known that the crystallization of semicrystalline polymers largely depends on the self-assembling of the nucleators. Knowing the abovementioned concentration (*c*)–temperature (*T*) diagram, the MSZ provides a mean for developing well-controlled crystallization of TMC-328. Therefore, in order to investigate the effect of the self-assembling of TMC-328 on the subsequent crystallization of PLLA, its isothermal crystallization was studied first. We first employed POM to observe the crystal structure of TMC-328 during crystallization at different temperatures selected from the MSZ or at its edges with the crystallization line for the PLLA-0.3 sample (Figure 3). The most intuitive observation from Figure 3 is that the higher the temperature, the clearer the fibrous structure is. Indeed, it is because the formation of fewer crystals of TMC-328 at high temperatures results in relatively thicker fibrils. On the other hand, the morphology of tightly packed fibrils of the nucleator is observed during isothermal crystallization at low temperatures, mainly because of the interconnection of many short little fibrils and their branches, displaying the network morphology.

**Figure 4:**
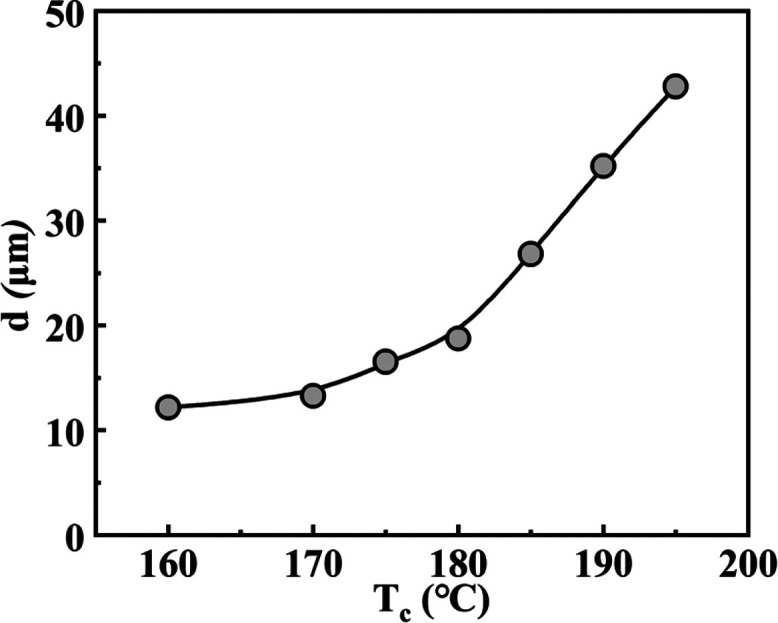
Evolution of the average distance between TMC-328 crystals in PLLA-0.3 as a function of crystallization temperature.

In order to quantify the visual differences in the self-assembling structure formed by TMC-328 during isothermal crystallization in the PLLA matrix at various temperatures, the average distance (*d*) of the adjacent fibrous crystals was calculated. The distance between the adjacent fibers of TMC-328 is calculated using an image analysis software (Image J), and it is the average of the interfiber distances at 100 randomly selected positions. The choice of this method to quantify the obtained morphology is the only possibility to quantify the obtained morphology, given that the high number of fibrils and their interconnection prevent the direct determination of the fibril concentration. This parameter assumes the physical meaning of the nucleation density or spatial density of the network structure. Figure 4 shows the *d* value of the adjacent fibrous crystals of TMC-328 in the PLLA-0.3 sample, which increases as the crystallization temperature increases, indicating that the higher the temperature, the lower is the nucleation density.

**Figure 5:**
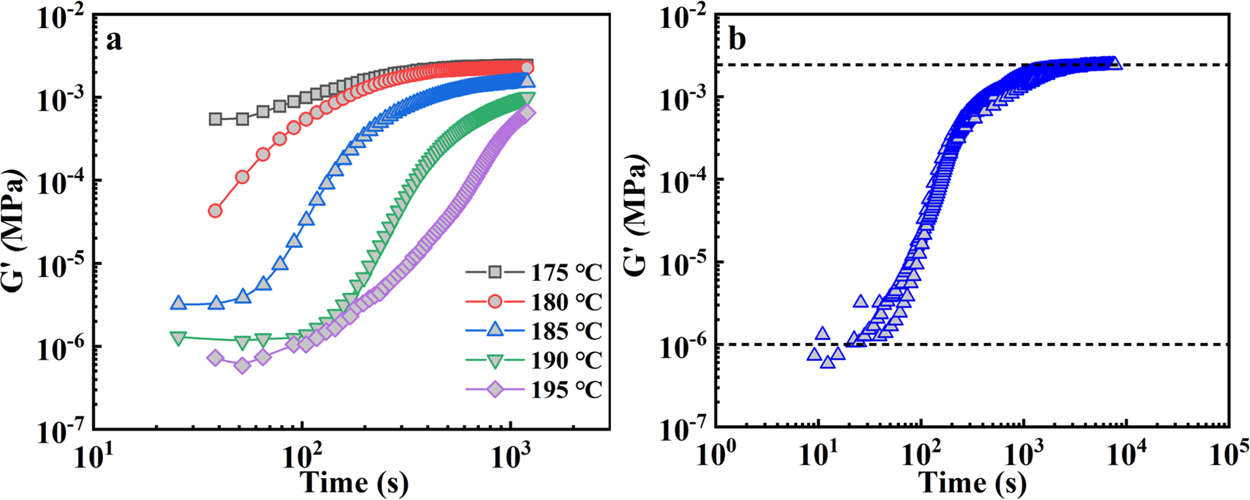
(a) Storage modulus, *G′*, as a function of time for TMC-328 crystallization in PLLA-0.3 samples at different temperatures. (b) Master curve of storage modulus with data at 185 °C taken as a reference, and the initial and final storage moduli are indicated by black dashed lines.

Having derived morphological information on the self-assembling/crystallization behavior of TMC-328, we now turn our attention to the phase separation kinetics. Rheology can be conveniently applied to follow the crystallization kinetics of TMC-328 in PLLA-0.3, especially because conventional differential scanning calorimetry (DSC) is nonapplicable, as the exceedingly low heat flow is below the sensitivity of the instrument. The evolution of storage modulus, *G′*, with time during isothermal crystallization for TMC-328 at different temperatures is shown in Figure 5a. The storage modulus starts from an initial plateau value and then rapidly increases by orders of magnitude with the growing crystallites of TMC-328 in the supersaturated solution, up to a higher plateau value, exhibiting a sigmoidal trend. The differences in the initial plateau modulus are related to the viscosity of the PLLA matrix at different isothermal crystallization temperatures, while the drop in the final plateau modulus with temperature increase is probably attributed to the reduced connectivity of the network structure or to the reduced volume fraction of fibrous crystals (see Figures 3 and 4). The initial storage modulus at 190 and 195 °C are similar, meaning that there is just a minor change in the viscosity in this temperature range. The similarity of storage moduli for the two tested temperatures at 200 s implies a similar structure of the fibrillar network under these conditions. In fact, the relationship between the final modulus and the self-assembling structure of TMC-328 in the PLLA matrix after crystallization is presented in Figure S3. An almost linear increase in the final modulus with the decrease in the average distance between the adjacent fibrils is found.

During isothermal crystallization, amorphous and crystalline phases within rheological measurements can be assumed to behave as two different viscoelastic elements. With the development of crystallization, the storage modulus continues to grow. Therefore, isothermal crystallization kinetics can be analyzed using the following equation:
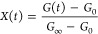
1where *X*(*t*) is the crystallinity (or the degree of transformation), and *G*_0_ and *G*_∞_ are the initial and final storage modulus, respectively. The half-crystallization time, *t*_0.5_, is defined as the time when the value of *X*(*t*) equals 0.5, and it is one of the most intuitive evaluation parameters for crystallization kinetics. Furthermore, a master curve of storage modulus at an arbitrary temperature taken as a reference, extending over a broader time range, was obtained with the software TRIOS. The master curve of storage modulus with crystallization data at 185 °C taken as a reference is shown in Figure 5b, and the corresponding shift factors (*f*_shift_) are summarized in the Supporting Information, Table S1. Therefore, the half-crystallization time for the nucleator at a chosen temperature (T*) can be calculated from the shift factor as follows: *t*_0.5_^*^(T*) = *t*_0.5_^185 ° C^ × *f*_shift (T*)_.

**Figure 6:**
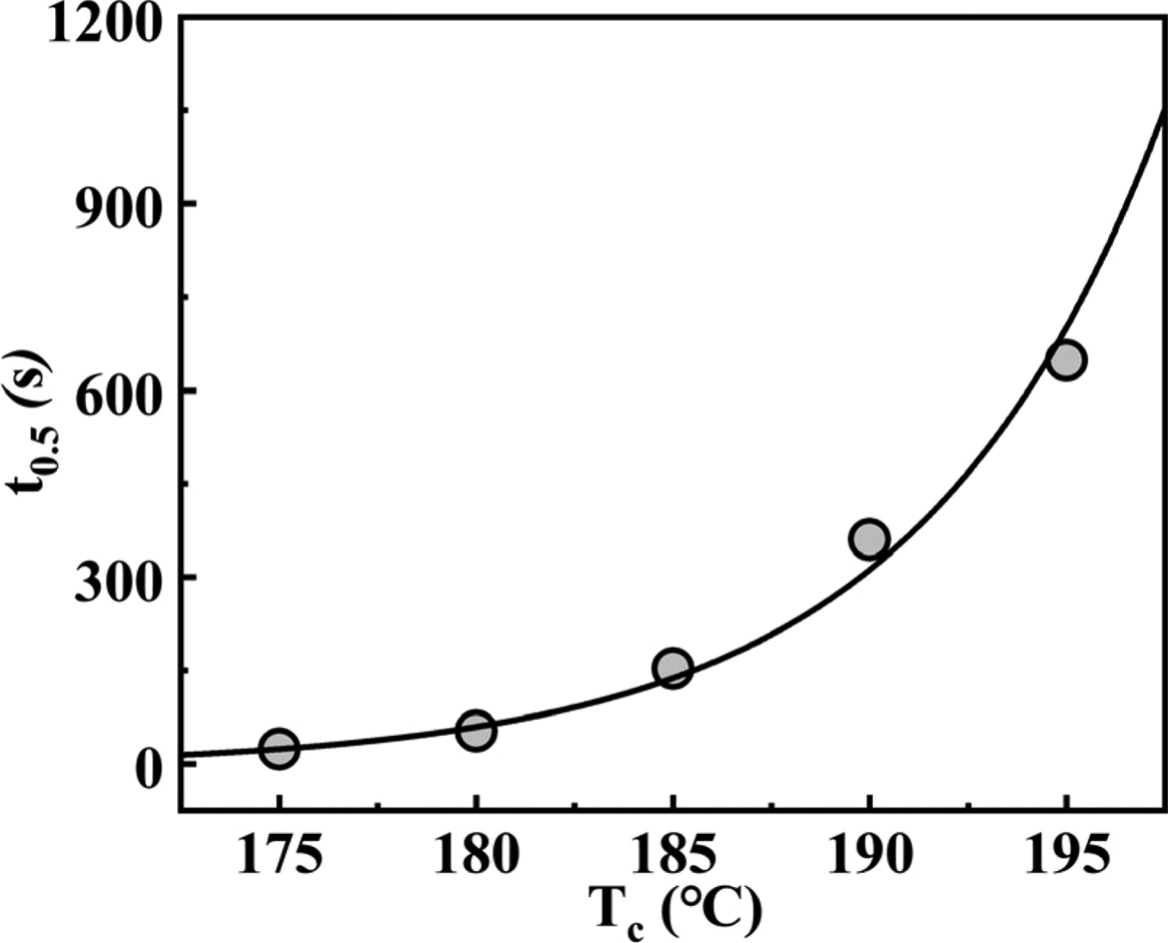
Half-crystallization time as a function of crystallization temperature for TMC-328 in the PLLA-0.3 sample melt.

The half-crystallization time for TMC-328 is plotted as a function of crystallization temperature in Figure 6. A decreasing trend of the crystallization rate with increasing temperature is observed, indicating that in the explored temperature range, the viscosity of the PLLA solvent is not the kinetically controlling factor for crystallization. Therefore, the crystallization kinetics of TMC-328 in this temperature range is dominated by a nucleation process.

### Relationship between Crystallization and Solubility of TMC-328 in the PLLA Matrix

It is known that the amount of nuclei (*N*) depends on both time (*t*) and supersaturation (Δ*c*) for crystallization in a solvent, that is, *N* = *f*(*t*, Δ*c*).^48,49^ The S-shaped dependence of the number densities of nuclei (i.e., from zero till the maximum number of nuclei) on time at fixed supersaturation has been determined earlier and verified in modern times. On the other hand, supersaturation is also a factor worth noticing because it is the driving force for crystal nucleation.

Indeed, the logistic (sigmoidal) functional description of nucleation kinetics can be used as a general physical rule of the nucleation process. This is because it just does not only apply to macromolecules and small inorganic molecules but is also valid for classical and two-stage (multistage) nucleation scenarios.^50,51^ In this case, a general formula describing the dependence of the nuclei density on time is proposed:
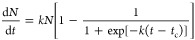
2where *k* is the formation frequency of critical nuclei, and *t*_c_ is the time of the sigmoid midpoint when *N* becomes half of the saturation nuclei number density (*N*_s_), i.e., *N* = *N*_s_/2.

To figure out the effect of supersaturation on nucleation kinetics in a solvent, Nývlt has proposed a semiempirical model to express the relationship between the nucleation rate and supersaturation from solutions at a constant cooling speed, which has been widely used:^43^
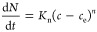
3where *K*_n_ is the nucleation constant, *c* and *c*_e_ are the actual solution concentration and the solubility, respectively. The exponent *n* is case-dependent.

Equalizing eqs 2 and 3, we obtain the following equation:
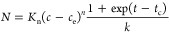
4

**Figure 7:**
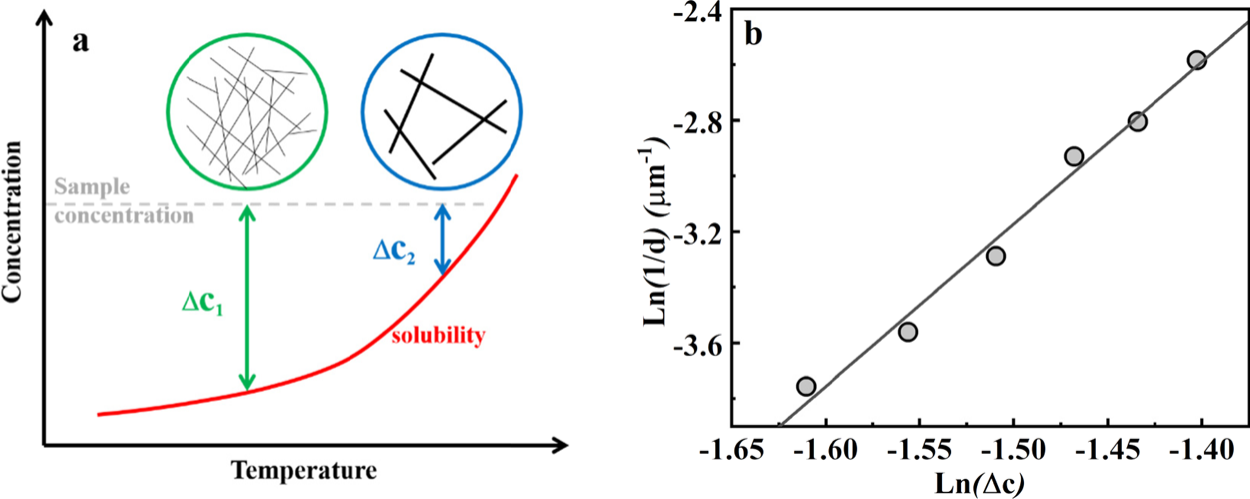
(a) Scheme of the effect of solubility on crystallization at different temperatures. (b) Evolution of number densities of fibrils of TMC-328 as a function of supersaturation at different temperatures.

It is notable that the number of growing crystals and their sizes are interrelated in a reciprocal manner in the process of crystallization. Based on eq 4, it is evident that for obtaining a small number of larger crystals (i.e., slower nucleation), it is necessary to choose a short nucleation time and minimum supersaturation, and vice versa, as indicated in the schematic of Figure 7a, which represents the effect of solubility on the crystallization of TMC-328 at different temperatures within a similar time frame. Because of the formation of extremely thin fibrils, it is hard to judge the crystal size in our work. Therefore, we use the average distance of adjacent crystals, assuming that it is inversely proportional to the crystal size and thus representative of the nuclei number density. From Figure 7a, we can see that with the increase in temperature, supersaturation decreases (Δ*c*_2_ < Δ*c*_1_) gradually for TMC-328, and then, the corresponding fibrils become larger and sparser (see Figure 3). The quantitative relationship between a parameter proportional to the nuclei number densities (1/*d*) and supersaturation is shown in Figure 7b, confirming that the nucleation density shows a strong dependence on supersaturation.

**Figure 8:**
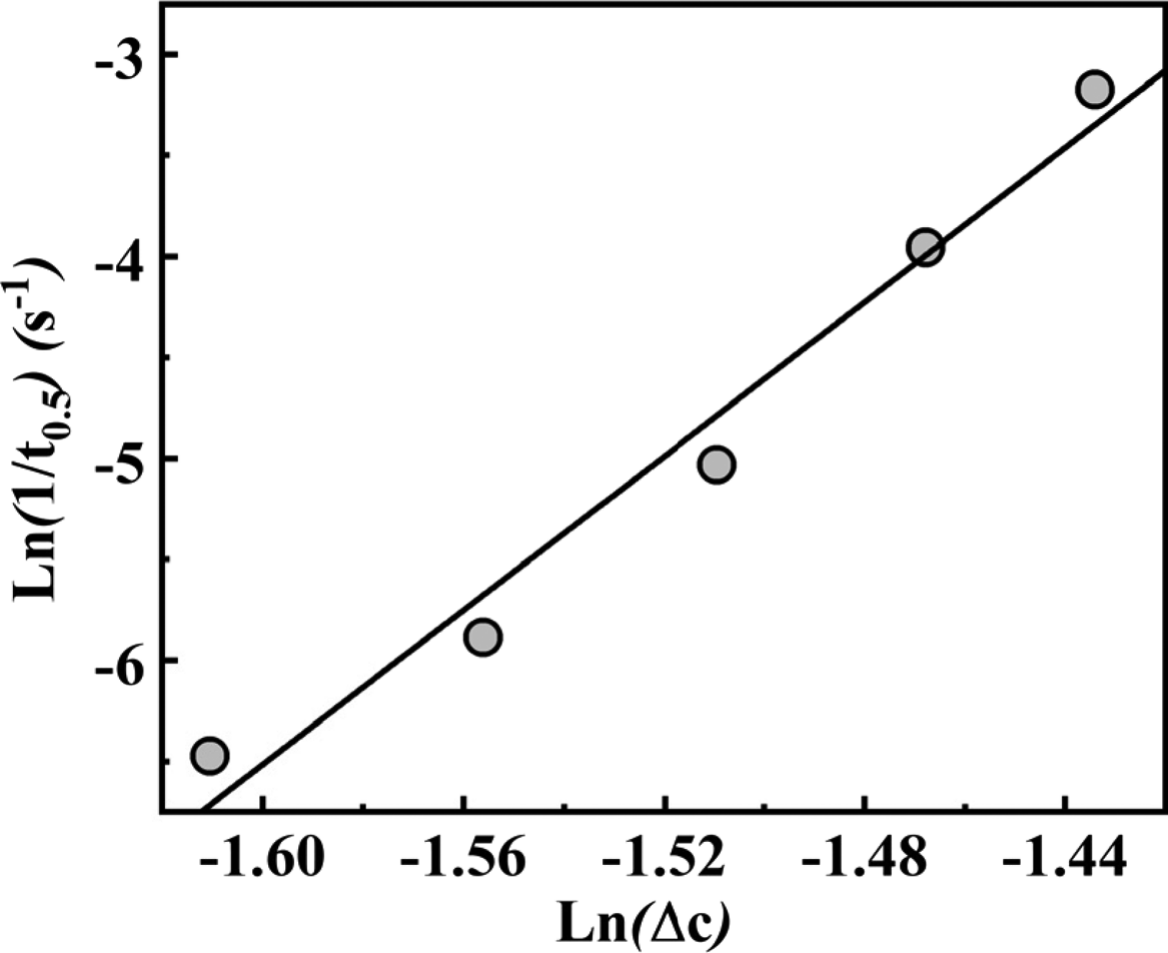
Reciprocal of the half-crystallization time of TMC-328 as a function of supersaturation at different temperatures.

Figure 8 shows the reciprocal of the half-crystallization time of TMC-328 at different temperatures as determined from rheology, as a function of supersaturation. Assuming the inverse half-crystallization time as a representative of the nucleation rate, the relationship shown in Figure 8 is in agreement with Nývlt’s equation, with an exponent *n* equal to 19.1. Indeed, as the formation of nuclei is faster, the larger is the driving force for precipitation.

Based on the classical nucleation theory, under the assumption of a spherical nucleus shape, the steady-state nucleation rate (*J*) in a solvent can be derived as follows:^52,53^
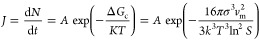
5where constant *A* is the pre-exponential factor, Δ*G_c_* is the activation energy of nucleation, *K* is the Boltzmann constant, σ is the interfacial free energy, *ν*_m_ is the solute molecular volume, which can be calculated using the method of atomic and bond contributions of van der Waals volume,^54^*T* is the crystallization temperature, and *S* is the supersaturation ratio (concentration *c* over solubility *c*_e_). Consequently, the critical nuclei size (r*), critical Gibbs free energy (ΔG*), and the number of molecules making up the nucleus (n*) can be determined as follows:^52,55^
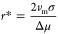
6
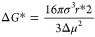
7
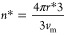
8where Δμ is the difference in the chemical potential between the solute in the solution and in the crystalline bulk phase, serving as the thermodynamic driving force for nucleation, which is usually estimated by the actual and equilibrium solute mole fraction in the solution, .

Usually, the induction time (*t*_i_) is proposed as the *t* representative of the nucleation rate (*J*), being inversely proportional to the nucleation rate in the solution of volume *V*:^56^
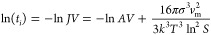
9

**Figure 9:**
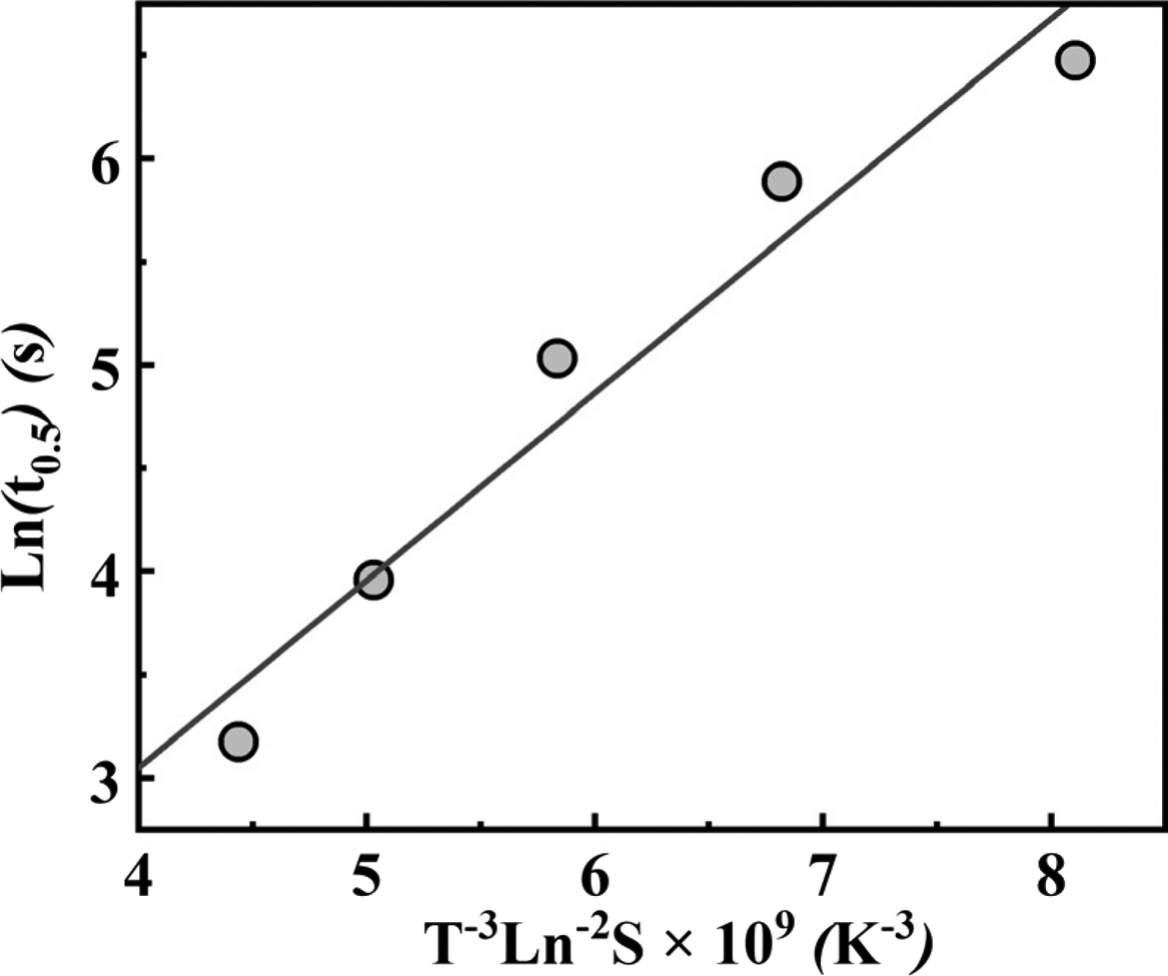
Half-crystallization time of TMC-328 in the PLLA matrix as a function of *T*^–3^ ln^–2^*S*.

For the crystallization of TMC-328 in the PLLA matrix, the overall crystallization kinetics is nucleation-controlled within the temperature range from 175 to 195 °C, as previously discussed. It seems reasonable to assume that the induction time is proportional to the half-crystallization time because in the POM micrograph, a distinct growth of the fibrils is not observed, but rather they appear at once. Therefore, *t*_0.5_ can be tentatively used as a parameter in eq 9 for the calculation of nucleation kinetics. Figure 9 shows the half-crystallization time of TMC-328 in the PLLA matrix as a function of *T*^–3^ ln^–2^*S*. A linear relationship is observed with a slope of 0.91 and an intercept of 0.58. The interfacial energy σ can be derived from the slope, and then, the corresponding critical parameters can be calculated and are summarized in Table 1.

**Table 1:** Values of the Supersaturation Ratio, Gibbs Free Energy, the Radius and Number of Molecules in a Critical Nucleus, for TMC-328 Crystallization in the PLLA Matrix at Different Temperatures

*T* (°C)	*S*	ΔG* (kJ/mol)	r* (nm)	n*
175	4.87	15.0	0.82	5.1
180	4.31	17.2	0.88	6.3
185	3.80	20.2	0.96	7.9
190	3.37	23.9	1.04	10.2
195	2.99	28.7	1.14	13.4

It is worth noting that an almost twofold change in the free energy barrier for nucleation occurs from a crystallization temperature of 175 to 195 °C, which is associated with an increase in the number of molecules involved in the critical size cluster from 5 to 13.

### Crystallization of PLLA Nucleated by TMC-328

For self-assembly systems, the hydrogen bond usually acts as the driving force to build superstructures because of its specificity and directionality.^31,57,58^ Based on this point, intensive research has been conducted, also including these self-assembly nucleators that have the acyl group, such as TMC-328, TMC-306, and TMC-300.^18,31,39,59−63^ Furthermore, the proton donors (NH) in the acyl group of nucleators are also expected to interact with the proton acceptors (C=O) in PLLA, promoting its crystallization. At the mesoscopic level, it is important to figure out the effect of the network morphology of the nucleator on the crystallization of PLLA.

**Figure 10:**
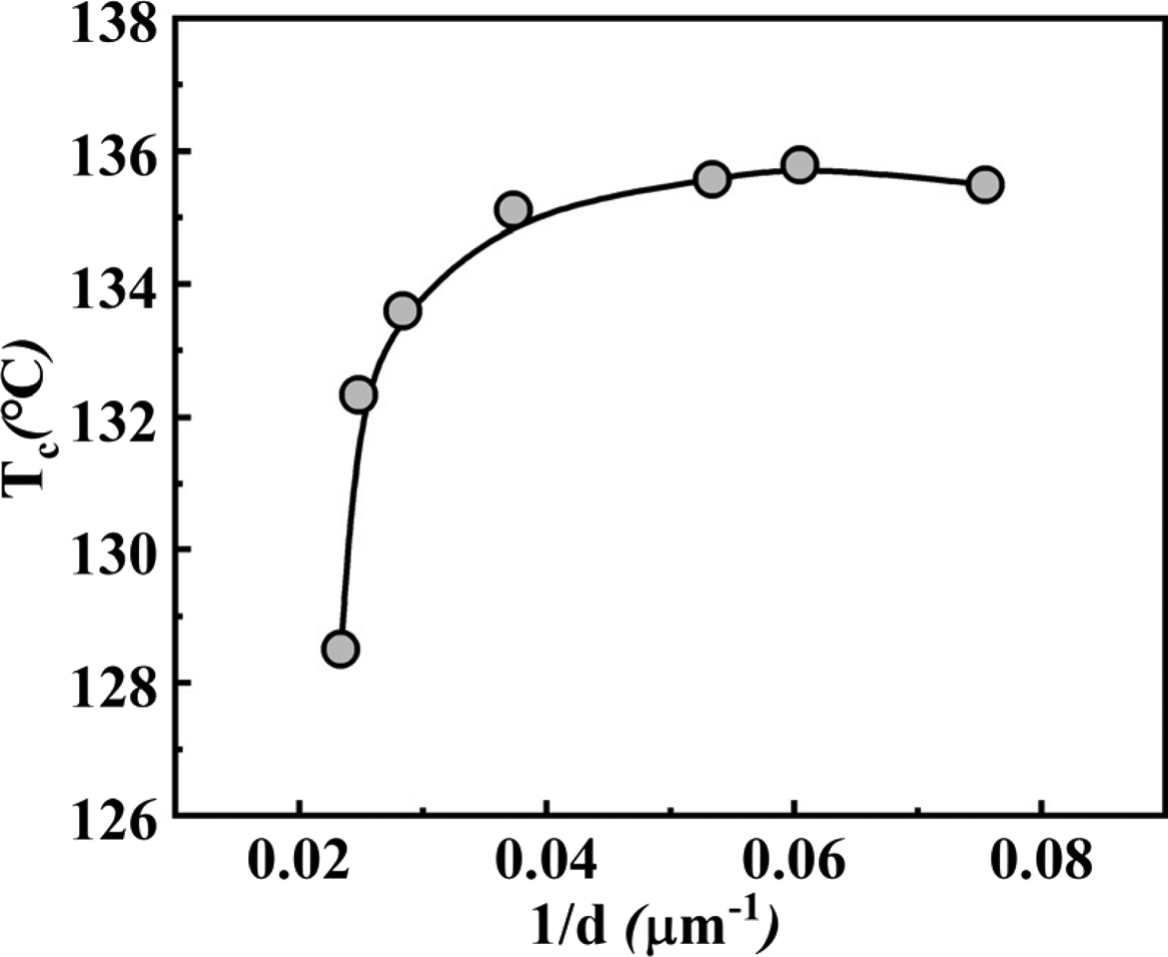
Crystallization temperature of PLLA upon cooling from the isothermal temperatures of TMC-328, as a function of the TMC-328 fibril spatial density in PLLA-0.3.

Figure 10 shows the evolution of the crystallization temperature of PLLA upon cooling from the temperatures where TMC-328 isothermally crystallizes as a function of the spatial density of fibrils, that is, their number per unit length (1/*d*) of TMC-328 in the PLLA matrix. When the fibril density becomes lower than about 0.04 μm^–1^, the crystallization rate of PLLA increases markedly (higher *T*_c_) as the network of fibrous crystals of TMC-328 becomes much denser and structured. Although the size of the crystal structure of TMC-328 is large in the high network formation temperature range (Figure 3), it is not efficient for the subsequent crystallization of PLLA, given the observed lower crystallization temperature of the polymer. Clearly, the crystallization of PLLA is related to the nucleating sites provided by the surface of fibrils of TMC-328. The higher the fibril density, the larger are the nucleating sites. On the other hand, the crystallization rate of PLLA does not change anymore when the nuclei density exceeds 0.04 μm^–1^ at network formation temperatures of TMC-328 below 185 °C. There may be various reasons to explain this observation. The crystallization temperature under nonisothermal conditions is influenced by both nucleation and growth processes. Therefore, even if there are many nucleation sites provided by the fibrillar structure of TMC-328 after isothermal crystallization at temperatures below 185 °C, the continuous growth of PLLA crystals becomes the rate-determining step because of the early-stage collision of extremely close crystals formed on the surface of dense fibrils. Another speculative explanation might refer to the “consumption” of hydrogen bond sites because of the strong intermolecular association within the self-assembling nucleator at lower temperatures. In fact, the intensity of the hydrogen bond for a given system generally increases with decreasing temperature because the enthalpy of bond formation is usually negative.^31^

**Figure 11:**
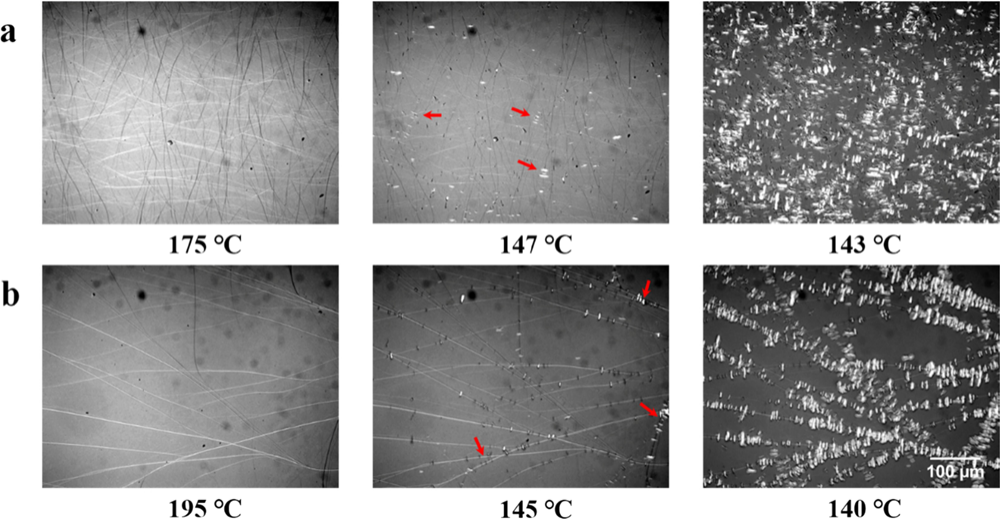
Polarized optical micrographs taken during cooling after self-assembling of TMC-328 at (a) 175 °C and (b) 195 °C for 20 min in PLLA-0.3.

To further understand the effect of TMC-328 on PLLA crystallization, the crystal morphologies of PLLA under the presence of various networks of the nucleator were characterized by polarized light microscopy, as shown in Figure 11. It has been shown that TMC-328 is randomly distributed in the form of an interconnected network structure formed by fibrils at low temperatures and of an isolated fibril structure at high temperatures in the PLLA melt (Figure 3). For both cases, disk-shaped kebabs of PLLA grow on the surface of the self-organized TMC-328 structures and form shish-kebab-like structures, which are indicated by the red arrows, and this result is in agreement with the previous studies.^35,36^ The final morphology of PLLA is similar after TMC-328 self-assembling at both temperatures (i.e., 175 and 195 °C, see Figure 11), notwithstanding the difference in the TMC-328 fibril spatial density. Several mechanisms have been proposed to explain the crystallization of PLLA with the aid of TMC-328, which include the epitaxial growth proposed by Bai et al.,^36^ and hydrogen bonding interactions proposed by Xie et al.^63^ This latter mechanism suggests that the absorbed PLLA chains onto the surface of self-organized fibrils of TMC-328 will form the primary nuclei, driven by hydrogen bonding interactions, and then, the outer layer molecules will crystallize on the surface of the primary nuclei when the temperature is further decreased. The interpretation has the support of Fourier-transform infrared (FT-IR) data, while the idea of epitaxial growth is still lacking direct evidence so far.

## Conclusions

The present work focuses on the isothermal crystallization of TMC-328 at different temperatures in a molten PLLA matrix and on the subsequent induced crystallization of PLLA. This nucleator presents a fibrous structure produced via self-assembling and develops into an interconnected network when its crystallization temperature is decreased. The crystal nuclei density of TMC-328 is quantified by means of the average distance (*d*) between adjacent fibrils, showing a steady decrease with the decrease in temperature. On the basis of the classical nucleation theory, the solid–melt interfacial energy, the size of the critical nucleus, and the number of molecules making up the critical nucleus of TMC-328 have been determined in the PLLA matrix for the first time, using the half-crystallization time obtained from rheological measurements.

The crystallization rate of PLLA is first largely enhanced with the increase in the TMC-328 fibril density because of the availability of a large nucleating surface. The acceleration then reaches a plateau with a further increase in the fibril spatial density because of the growth stage becoming the rate-determining step. Notwithstanding the nucleation density of the nucleator, the morphology after the crystallization of PLLA is the hybrid shish-kebab structure, which is in agreement with previous studies.

## Supporting Information Available

Chemical structure of the NA; TGA scans for PLLA and TMC-328; final storage modulus of PLLA-0.3 as a function of the reciprocal of the average distance (1/*d*) of the adjacent TMC-328 fibrils; and the shift factor of storage modulus versus time curves at different temperatures (PDF)
