# Supplementary material for: Crystallization of a Self-Assembling Nucleator in Poly(l-lactide) Melt
Source: Cryst Growth Des. 2021 Sep 1;21(10):5880–8. doi: 10.1021/acs.cgd.1c00750 (PMC8499044; doi:10.1021/acs.cgd.1c00750)
Supplement: Supplementary file 1 — cg1c00750_si_001.pdf [file cg1c00750_si_001.pdf]

Crystallization of a self-assembling nucleator in poly(L-lactide) melt

Wei Wang,[a] Angelo Saperdi,[a] Andrea Dodero,[a] Maila Castellano,[a] Alejandro J. Müller,[b, c] Xia Dong,[d, e] Dujin Wang, [d, e] Dario Cavallo*, [a]

[a] Department of Chemistry and Industrial Chemistry, University of Genoa, Via Dodecaneso 31, 16146 Genova, Italy

[b] POLYMAT and Department of Polymers and Advanced Materials: Physics, Chemistry and Technology, Faculty of Chemistry, University of the Basque Country UPV/EHU, Paseo Manuel de Lardizabal, 3, 20018 Donostia—San Sebastian

[c] IKERBASQUE, Basque Foundation for Science, 48009 Bilbao, Spain

[d] Beijing National Laboratory for Molecular Sciences, Institute of Chemistry, Chinese Academy of Sciences, Beijing, 100190, China

[e] University of Chinese Academy of Sciences, Beijing, 100049, China

*Corresponding Authors: (D.C.) dario.cavallo@unige.it

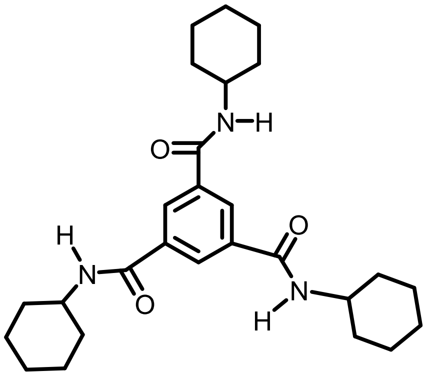

Figure S1. Chemical structure of nucleating agent TMC-328.

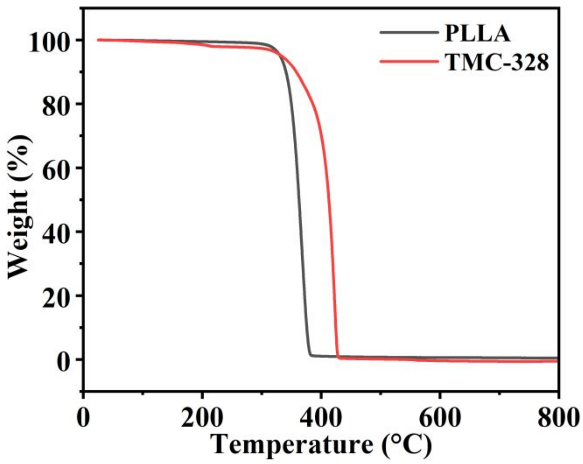

Figure S2. Thermogravimetric analysis (TGA) measurements for the PLLA and TMC-328.

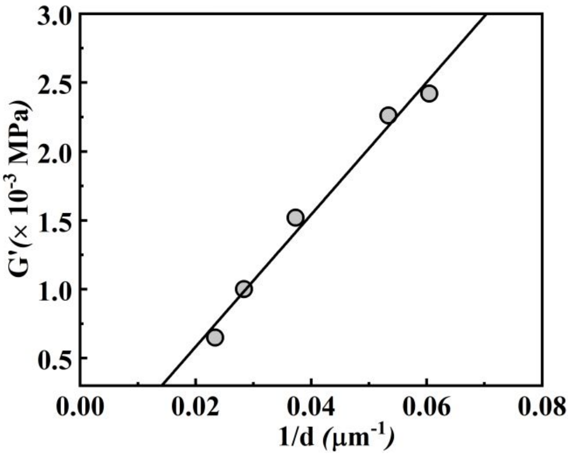

Figure S3. Final storage modulus of PLLA-0.3 as a function of the reciprocal of average distance (1/d) of adjacent fibrils of TMC-328.

Table S1. Shift factor of storage modulus versus time curve at different temperatures.

T (°C)	f_{shift}
175	0.156
180	0.341
185	1.000
190	2.350
195	4.233
